# Shared and Distinct Functions of the Transcription Factors IRF4 and IRF8 in Myeloid Cell Development

**DOI:** 10.1371/journal.pone.0025812

**Published:** 2011-10-07

**Authors:** Michio Yamamoto, Takayuki Kato, Chie Hotta, Akira Nishiyama, Daisuke Kurotaki, Masahiro Yoshinari, Masamichi Takami, Motohide Ichino, Masatoshi Nakazawa, Toshifumi Matsuyama, Ryutaro Kamijo, Seiichi Kitagawa, Keiko Ozato, Tomohiko Tamura

**Affiliations:** 1 Department of Immunology, Yokohama City University Graduate School of Medicine, Yokohama, Japan; 2 Department of Physiology, Osaka City University Graduate School of Medicine, Osaka, Japan; 3 Department of Biochemistry, School of Dentistry, Showa University, Tokyo, Japan; 4 Department of Experimental Animal Science, Yokohama City University Graduate School of Medicine, Yokohama, Japan; 5 Department of Molecular Microbiology and Immunology, Graduate School of Biomedical Sciences, Nagasaki University, Nagasaki, Japan; 6 Program in Genomics of Differentiation, Eunice Kennedy Shriver National Institute of Child Health and Human Development, National Institutes of Health, Bethesda, Maryland, United States of America; Emory University, United States of America

## Abstract

Interferon regulatory factor (IRF) 8 and IRF4 are structurally-related, hematopoietic cell-specific transcription factors that cooperatively regulate the differentiation of dendritic cells and B cells. Whilst in myeloid cells IRF8 is known to modulate growth and differentiation, the role of IRF4 is poorly understood. In this study, we show that IRF4 has activities similar to IRF8 in regulating myeloid cell development. The ectopic expression of IRF4 in myeloid progenitor cells *in vitro* inhibits cell growth, promotes macrophages, but hinders granulocytic cell differentiation. We also show that IRF4 binds to and activates transcription through the IRF-Ets composite sequence (IECS). Furthermore, we demonstrate that *Irf8* ^-/-^ *Irf4* ^-/-^ mice exhibit a more severe chronic myeloid leukemia (CML)-like disease than *Irf8* ^-/-^ mice, involving a disproportionate expansion of granulocytes at the expense of monocytes/macrophages. *Irf4* ^-/-^ mice, however, display no obvious abnormality in myeloid cell development, presumably because IRF4 is expressed at a much lower level than IRF8 in granulocyte-macrophage progenitors. Our results also suggest that IRF8 and IRF4 have not only common but also specific activities in myeloid cells. Since the expression of both the *IRF8* and *IRF4* genes is downregulated in CML patients, these results may add to our understanding of CML pathogenesis.

## Introduction

Cell differentiation requires appropriate changes in gene expression patterns, which are tightly regulated by cell type-specific transcription factors. In case of hematopoiesis, dysregulation of these processes can result in hematopoietic disorders such as leukemias [1]. Myeloid progenitor cells, defined as granulocyte-macrophage progenitors (GMPs) [2], give rise to granulocytes (such as neutrophils) or monocytes/macrophages. A number of transcription factors including PU.1, C/EBPs and Interferon Regulatory Factor 8 (IRF8) have been shown to regulate this process. While PU.1 is essential for macrophage differentiation in particular, C/EBPα and C/EBPε are the critical drivers of granulocyte differentiation [3]. We have shown previously that IRF8, a hematopoietic cell-specific factor belonging to the IRF family, directs macrophage differentiation whilst it inhibits myeloid cell growth and neutrophil differentiation [4], [5]. We have also previously identified an IRF8's target DNA element termed the IRF-Ets Composite Element (IECS; represented by GAAANN[N]GGAA) and multiple direct target genes including those encoding Blimp-1, Cathepsin C and Cystatin C [6], [7]. Importantly, mice lacking the *Irf8* gene (*Irf8* ^-/-^ mice) develop a chronic myelogenous leukemia (CML)-like syndrome, in which there is a disproportionate expansion of neutrophils at the expense of monocytes/macrophages [8], [9], [10]. Furthermore, cells from human CML patients lack the expression of *IRF8* [11], suggesting that its loss is a key aspect of human CML pathogenesis. Conservation of IRF8's function between mice and humans has been proven by a recent study demonstrating that a loss-of-function mutation in the human *IRF8* gene also results in a very high neutrophil count and an absence of circulating monocytes and dendritic cells [12].

IRF4 is another hematopoietic cell-specific IRF and has the highest amino acid sequence similarity with IRF8. Consistent with this structural similarity, both IRFs have an ability to interact with the Ets transcription factor PU.1, required also for B cell differentiation, and to activate transcription via the Ets-IRF Composite Element (EICE; GGAANNGAAA) [13]. The EICE is another DNA sequence targeted by IRF and PU.1, and is active at the promoters of B cell-specific genes such as immunoglobulin light chain genes. In fact, IRF4 and IRF8 are expressed in B lineage cells and cooperatively stimulate the development of B cells [14]. In dendritic cells (DCs) consisting of multiple subsets, these two IRFs are expressed in a subset-selective manner and govern the generation of corresponding subsets [15], [16] via their common and specific activities [16]. It has been reported also that IRF4, like IRF8, is expressed in macrophages [17]. However, the role of IRF4 in myeloid cell development remains poorly understood. In our current study, we have examined whether IRF4 has any roles in regulating myeloid cell growth and differentiation through gene introduction experiments and through the analysis of mice lacking *Irf4* and/or *Irf8*.

## Materials and Methods

### Ethics statement

All animal experimentations were conducted in accordance with NIH and Public Health Service (PHS) policy or the Guidelines for Proper Conduct of Animal Experiments (Science Council of Japan), and all protocols were approved by Eunice Kennedy Shriver NICHD Animal Care and Use Committee (Protocol #08-010) or institutional review boards in Yokohama City University (Protocol #09-127, 10-122).

### Cells, retroviral vectors, retroviral transduction and mice

Tot2 and 32Dcl.3 cells were cultured as described previously in the presence of granulocyte-macrophage colony stimulating factor (Peprotech) at 2.5 ng/ml and 10% WEHI3B-conditioned medium as a source of interleukin-3 (IL-3), respectively [4]. When inducing neutrophil differentiation in 32Dcl.3 cells, granulocyte-colony stimulating factor (G-CSF, Peprotech) was used at 10 ng/ml. To generate pMSCV-puro (Clontech) and pMSCV-CD8t [7] vectors carrying IRF8FLAG or IRF4FLAG, the FLAG peptide sequence was added to the 3′ side of *Irf4* or *Irf8* cDNA before the stop codon by PCR using Pfu DNA polymerase. The resulting fragments were then inserted into the vectors. pSIRV-IECS-Ld40-GFP has been described previously [7]. pSIRV-mIECS-Ld40-GFP was constructed by inserting three copies of a mutant IECS fragment (GAAACAGGGAA to GCTGCAGGGAA) into pSIRV-GFP. The nucleotide sequences of all constructs were confirmed by sequencing. Retroviral preparation and transduction were performed as described previously [7]. Transduced cells were purified by puromycin treatment (2 µg/mL) or immunomagnetic cell sorting. *Irf8* ^-/-^, *Irf4* ^-/-^, and *Irf8* ^-/-^ *Irf4* ^-/-^ mice in a C57BL/6 background were described previously [16] and used at 7 to 9 weeks of age.

### Quantitative RT-PCR

Total RNA was prepared using RNAiso Plus (Takara Bio), treated with DNase I (Invitrogen), and reverse transcribed using Primescript (Takara Bio) in accordance with the manufacturer's instructions. Quantitative PCR (qPCR) was performed in triplicate using the THUNDERBIRD SYBR qPCR Mix (Toyobo) and the ABI 7500 or StepOnePlus real-time PCR systems (Applied Biosystems) according to the manufacturers' protocols. The following primers were used: *Msr1* (sense, 5′-ATC ACC AAC GAC CTC AGA CT-3′; antisense, 5′-CCG ATC ACC TTT AAC ACC T-3′), *Irf5* (sense, 5′-ATG TCC TGG ACC GTG GGC TC-3′; antisense, 5′-GAA CAC CTT ACA CTG GCA CAG ACG-3′), *Mrc1* (sense, 5′-AGC CCA CAC CTG CTC CAC AAG A-3′; antisense, 5′-GCT CGC GCG TTG TCC ATG GTT-3′), *Il12b* (sense, 5′-GAC ACG CCT GAA GAA GAT GAC-3′; antisense, 5′-TAG TCC CTT TGG TCC AGT GTG-3′), *Il6* (sense, 5′-AGT TGC CTT CTT GGG ACT GA-3′; antisense, 5′-TCC ACG ATT TCC CAG AGA AC-3′), *Csf3r* (sense, 5′-CCC ACC ATC ATG ACA GAG-3′; antisense, 5′-CAG TGG GTC GGT TTC TTG T-3′), *Irf4* (sense, 5′-TCG GCC CAA CAA GCT AGA AA-3′; antisense, 5′-GGC CAT GGT GAG CAA ACA CT-3′), *Irf8* (sense, 5′-ATA TGC CGC CTA TGA CAC ACA CC-3′; antisense, 5′-TTG CCC CCG TAG TAG AAG CTG A-3′). The primers used to analyze the other genes tested in this study were as previously described [6], [7], [16]. Each of the primer sets produced a unique product. Data were analyzed using either the ΔΔCT method or the standard curve method, and normalized against the *Gapdh* expression levels.

### Flow cytometry

For cell-surface marker analysis, cells were stained as described previously [16] with the appropriate antibodies and analyzed by FACSCalibur or FACSCanto II (BD Biosciences). A phagocytosis assay was performed using Vybrant Phagocytosis Assay Kit (Invitrogen). In this experiment, the cells were incubated with fluorescein-labeled *E. coli* K-12 bioparticles at 37°C for 2 h, then washed twice before analysis by flow cytometry. As a control reaction, cells were incubated at 4°C. The resulting data were analyzed using the FlowJo software (TreeStar). For cell cycle analysis, cells were fixed in cold 70% ethanol, treated with 100 ng/mL RNaseA, and stained with 50 µg/mL propidium iodide. Stained cells were analyzed by FACSCalibur and cell cycle profiles obtained by using CellQuest and ModFitLD V2.0 software (BD Biosciences). Cell sorting was performed using FACSAria II (BD Biosciences) or MoFlo (Dako Cytomation) into the following lineages: common myeloid progenitors (CMPs), Lin^−^ (lineage marker-negative, CD5^−^ B220^−^ CD11b^−^ Gr1^−^ 7/4^−^Ter119^−^), IL-7Rα^−^ c-Kit^+^ Sca-1^−^ CD34^+^ FcγRII/III^−^; GMPs, Lin^−^ IL-7Rα^−^ c-Kit^+^ Sca-1^−^ CD34^+^ FcγRII/III^+^; granulocytes, CD11c^−^ CD11b^+^ F4/80^−^ Gr1^+^; T cells, CD3^+^; B cells, CD19^+^; plasmacytoid DCs (pDCs), CD11c^+^ PDCA-1^+^ B220^+^; and classical DCs (cDCs), CD11c^+^ PDCA-1^−^ B220^−^. CMPs and GMPs were obtained from bone marrow. Lin^−^ cells were enriched by the Lineage Cell Depletion Kit and the AutoMACS cell separation system (Miltenyi Biotec). Resident macrophages were obtained from the peritoneal cavity and purified using plastic plate adherence. Other cell types were obtained from the spleen. The purity of the sorted cells was >95%. 7-Amino-Actinomycin D (eBioscience) was used to exclude dead cells. Antibodies were purchased from BD Pharmingen, eBioscience or BioLegend.

### Reporter Assay

Reporter assays were performed as described previously [7] with slight modifications. Briefly, cells were transduced with SIRV-IECS-Ld40-GFP or SIRV-mIECS-Ld40-GFP reporter constructs, selected with puromycin, and then transduced with MSCV-CD8t vectors harboring IRF4FLAG or IRF8FLAG. Transduced cells were stained with anti-human CD8 conjugated with Cy-Chrome (BD Pharmingen). The promoter activities were analyzed using a FACSCanto II on day 2 after the transduction of MSCVs to acquire GFP signals in CD8^+^ cells. The data were analyzed using FlowJo software (TreeStar).

### Chromatin immunoprecipitation assay

Chromatin immunoprecipitation (ChIP) assays were performed as described previously [7] with slight modifications. Briefly, cell lysates were sonicated six times for 30 sec each at 1 min intervals using Bioruptor (Cosmo Bio) to shear the genomic DNA into 200- to 1000-base pair fragments. Immunoprecipitations were then performed using 2 µg of normal goat immunoglobulin G (IgG), goat anti-IRF8 antibody (C-19; Santa Cruz Biotechnology) or goat anti-IRF4 antibody (M-17; Santa Cruz Biotechnology). The primers used for qPCR were as described previously [7]. Data were analyzed using the ΔΔCT method.

## Results

### IRF4 induces macrophage differentiation and cell cycle arrest

**Figure 1:**
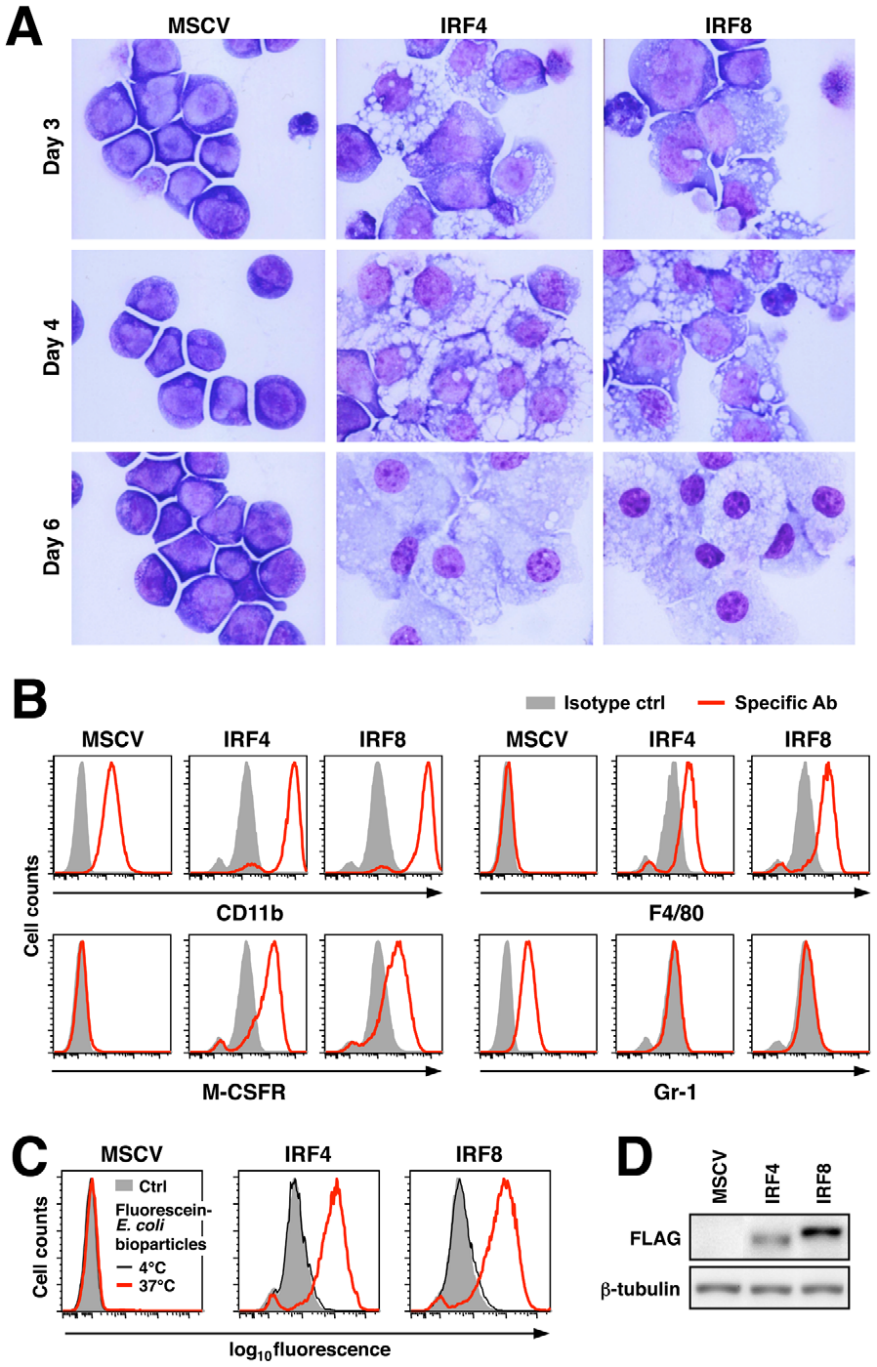
IRF4 drives the differentiation of myeloid progenitors towards macrophages. (A) Wright-Giemsa stain of Tot2 cells transduced with empty MSCV-puro, MSCV-IRF4FLAG-puro or MSCV-IRF8FLAG-puro (original magnification, x 600). (B) Surface marker analysis. Cells on day 6 were stained with the indicated antibodies or isotype control antibodies and analyzed by flow cytometry. Note that differentiated macrophages have higher autofluorescence than undifferentiated cells. (C) Phagocytic activity. Cells on day 6 were incubated with fluorescein-labeled *E. coli* bioparticles at 37°C or 4°C for 2 h and analyzed by flow cytometry. (D) Immunoblotting analysis of FLAG-tagged IRFs. β-tubulin expression is shown as a loading control.

We have previously shown that the introduction of IRF8 into *Irf8* ^-/-^ myeloid progenitor cell lines such as Tot2 causes their differentiation into mature macrophages concomitant with a cell cycle arrest at the G_0_/G_1_ phase. To examine whether IRF4 has a similar function, we introduced this factor into Tot2 cells using a MSCV retrovirus (MSCV-IRF4FLAG-puro). As shown in Figure 1A, IRF4-transduced cells manifested morphologic changes typical of macrophages, i.e., enlargement of the cytoplasm filled with vacuoles, and shrinkage and condensation of nucleus by day 6, which were similar also to those observed in cells transduced with IRF8-FLAG. Flow cytometry analysis demonstrated that these morphological changes accompanied the expression of CD11b, F4/80 and M-CSF receptor, and also the loss of Gr-1 expression (Figure 1B). Furthermore, IRF4-transduced cells exhibited strong phagocytic activity that was comparable to IRF8-transduced cells, whilst control MSCV-transduced cells did not show this activity (Figure 1C). These results indicate that the transduction of IRF4 or IRF8 cells results in their differentiation into bona fide macrophages. Immunoblotting analysis using an anti-FLAG antibody showed that the expression level of the introduced IRF4 was considerably lower than that of IRF8, suggesting a comparable or greater ability of IRF4 than IRF8 to cause morphological macrophage differentiation (Figure 1D). Of note, Tot2 cells do not express detectable levels of endogenous *Irf4* by semi-quantitative RT-PCR (data not shown), which would explain the strong dependence of this cell line on exogenous IRF8 or IRF4 for macrophage differentiation (also see below for the detection of *Irf4* expression in GMPs using a more sensitive qRT-PCR method).

**Figure 2:**
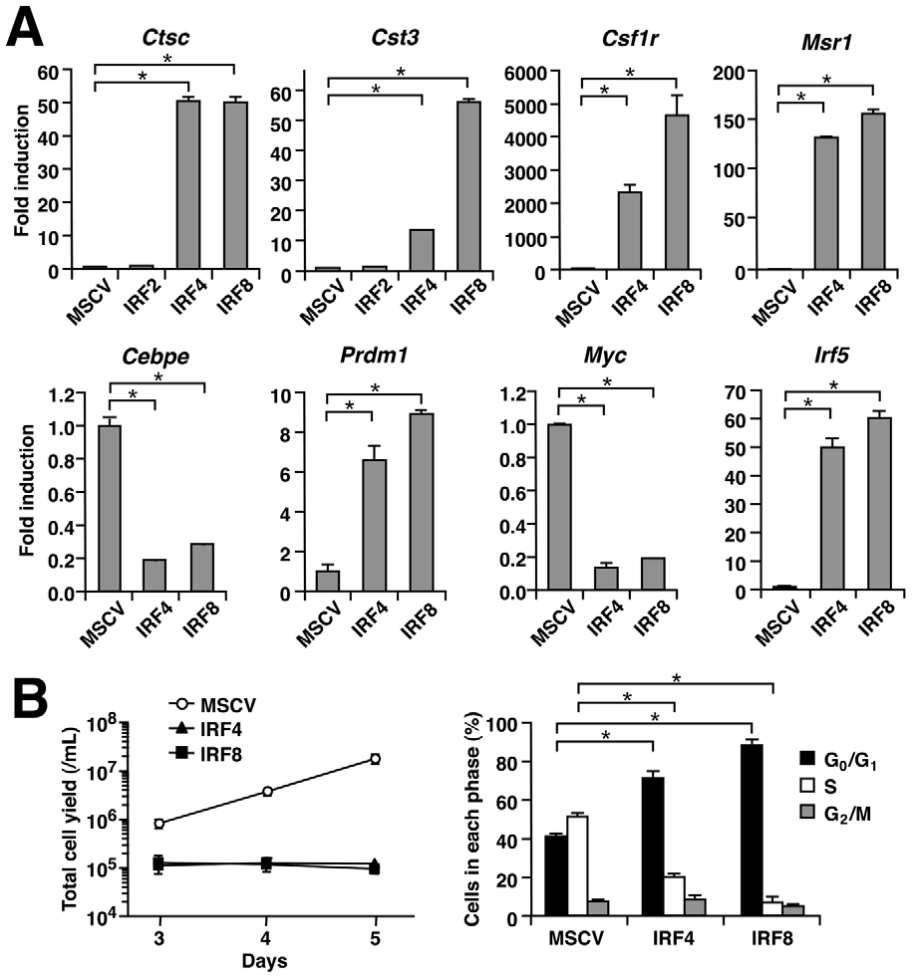
IRF4 induces macrophage-related genes and growth arrest during macrophage differentiation. (A) Induction of macrophage-related genes. Transcript levels in MSCV-transduced cells on day 5 were analyzed by qRT-PCR in triplicate. Data were analyzed using the ΔΔCT method and normalized by the *Gapdh* levels and shown as values relative to those in empty vector-transduced cells (mean ± standard deviation; representative of three independent experiments with similar results). *P<0.01 (Student's *t*-test). (B) Total viable cell yields (left panel) and cell cycle profiles (on day 4, right panel) after the transduction of MSCVs. Data are expressed as mean ± standard deviation of three independent experiments. *P<0.01 (Student's *t*-test).

qRT-PCR analysis revealed that various macrophage-related genes such as those encoding Cathepsin C (*Ctsc*), Cystatin C (*Cst3*), CSF-1 receptor/M-CSF receptor (*Csf1r*), Scavenger receptor (*Msr1*) and Blimp-1 (*Prdm1*), known to be induced by IRF8 [6], [7], are also strongly induced upon the introduction of IRF4, whereas IRF2 failed to induce *Ctsc* or *Cst3* (Figure 2A). On the other hand, both IRF4 and IRF8 inhibited the expression of the *Cebpe* gene that encodes C/EBPε, a transcription factor essential for neutrophil differentiation. In the case of *Cst3*, its induction by IRF4 was somewhat weaker than that by IRF8 but was still reproducibly detectable. Blimp-1 is a transcription factor that represses the *Myc* gene. *Myc* expression was in fact suppressed, and the cell cycle largely arrested at G_0_/G_1_, in cells transduced with IRF4 or IRF8 (Figure 2B). Interestingly, we further found that IRF4 and IRF8 induce the gene encoding IRF5, another IRF that is preferentially expressed in immune cells including macrophages (see below).

These results indicate that IRF4 has abilities that are similar to IRF8 in directing myeloid progenitor cells to differentiate towards macrophages and inducing cell growth arrest.

### Activation of IECS-mediated transcription by IRF4

**Figure 3:**
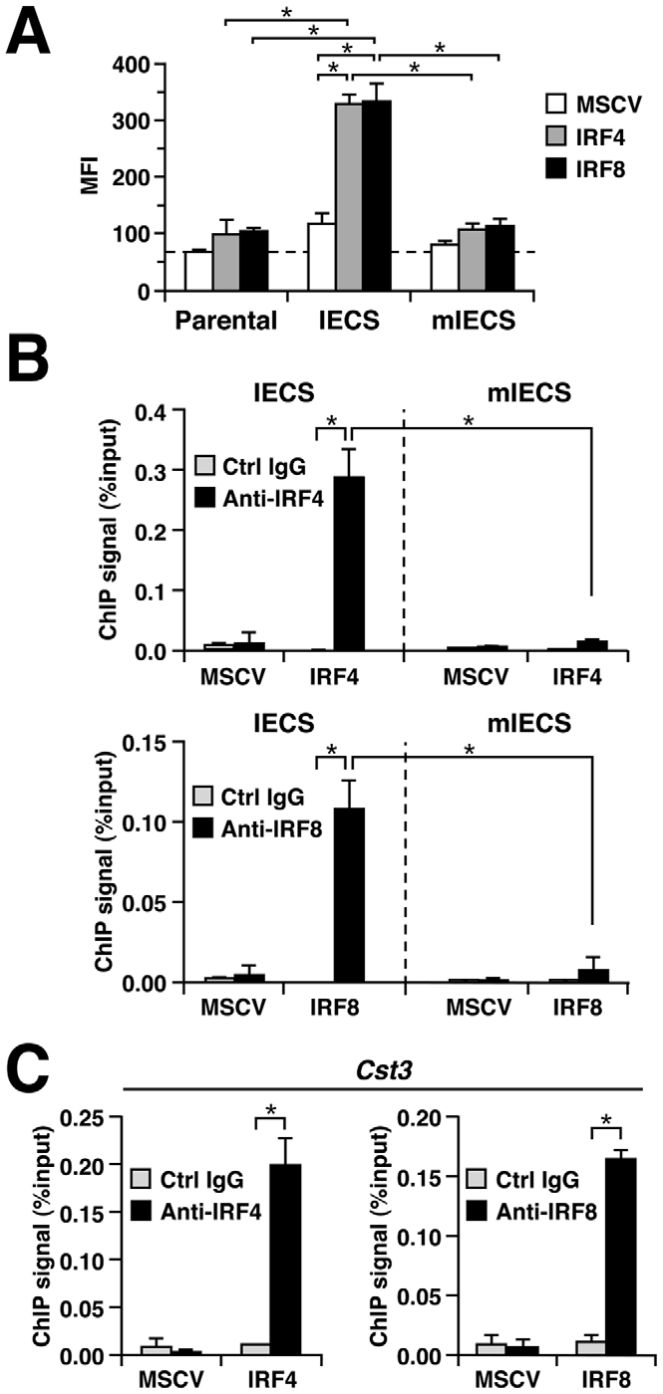
IRF4 targets the IECS. (A) Reporter assays of transcription through the IECS. Tot2 cells were transduced with SIRV-IECS-Ld40-GFP or SIRV-mIECS-Ld40-GFP and then with empty MSCV-CD8t, MSCV-IRF4-CD8t, or MSCV-IRF8-CD8t. The promoter activities in CD8^+^ cells were analyzed on day 2 after the transduction of MSCVs. The activity is shown as mean fluorescent intensity (MFI) of GFP signals (mean ± standard deviation of three independent experiments). *P<0.01 (Student's *t*-test). (B, C) ChIP assays for binding to the IECS (B) or a gene promoter containing an IECS (C). Cells transduced with SIRVs and MSCVs were analyzed by ChIP assays on day 3 after the transduction of MSCVs. Chromatin was precipitated by anti-IRF4 antibody, anti-IRF8 antibody, or normal goat IgG. Precipitated DNA was analyzed by qPCR in triplicate using primers that amplified the IECS sequence in SIRVs or those that amplified the IECS region of the *Cst3* gene promoter (mean ± standard deviation). Data are representative of three independent experiments. *P<0.01 (Student's *t*-test).

To next examine the mechanism by which IRF4 induces macrophage differentiation, we analyzed whether IRF4 targets the IECS, which was originally identified as the DNA element targeted by IRF8 in differentiating macrophages [7]. We first performed reporter assays using the self-inactivating retrovirus-based reporter, SIRV-GFP [7]. Tot2 cells were first transduced with SIRV-IECS-Ld40-GFP (in which GFP transcription is driven by three copies of the IECS from *Cst3*, a direct target gene of IRF8, followed by the minimal promoter Ld40) or SIRV-mIECS-Ld40-GFP (in which the core IRF binding sequence GAAA in the IECS is mutated), and further transduced with IRF4 or IRF8. The promoter activity levels were then quantified by measuring the GFP expression levels. The results showed that both IRFs strongly induced transcription via the IECS but not the mIECS containing promoters (Figure 3A).

We next performed chromatin ChIP assays using the same system, i.e. Tot2 cells transduced with SIRVs and IRFs. As shown in Figure 3B, both IRF4 and IRF8 bound to the IECS but not mIECS within the SIRV reporter cassette. As expected, this binding was not observed in empty MSCV-transduced cells. Likewise, both IRF4 and IRF8 bound to the endogenous *Cst3* gene promoter that contains an IECS (Figure 3C). Thus, IRF4 and IRF8 show a common ability to bind to, and thereby activate transcription, via the IECS.

### Specific activities of IRF4 and IRF8

**Figure 4:**
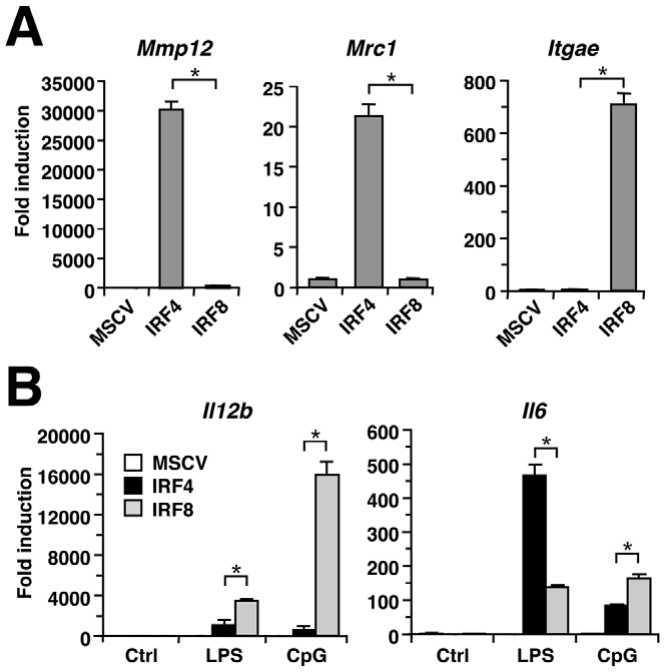
Specific activities of IRF4 and IRF8 in macrophages. (A) Genes specifically induced by IRF4 or IRF8. Tot2 cells transduced with empty MSCV-puro, MSCV-IRF4FLAG-puro or MSCV-IRF8FLAG-puro were analyzed by qRT-PCR on day 3 (mean ± standard deviation). Data are representative of two independent experiments with similar results. (B) Distinct patterns of cytokine gene induction in IRF4- and IRF8-transduced macrophages. Cells transduced with MSCVs were stimulated on day 5 with 1 µg/ml LPS or 1 µg/ml CpG-B for 5 h, and analyzed by qRT-PCR using the ΔΔCT method (mean ± standard deviation). Two repeat experiments gave similar results. *P<0.01 (Student's *t*-test).

We wished to next determine whether the activities of IRF4 and IRF8 in myeloid cells are equivalent. To address this question, we screened the expression of various genes in Tot2 cells 3 days after the transduction of IRF4, IRF8, or an empty vector. The Tot2 system was used in order to compare the activities of these IRFs in an early phase of macrophage differentiation on the same platform. Although many macrophage-related genes are induced by both IRF4 and IRF8 as shown in Figure 2A, we found that there are genes specifically induced by only one of these two IRFs. For example, the *Mmp12* and *Mrc1* genes, encoding matrix metalloproteinase-12 and the CD206/mannose receptor and CD169, respectively, were found to be specifically induced by IRF4, whereas *Itgae* (encoding integrin αE) was only activated by IRF8 (Figure 4A). These results suggest that IRF4 and IRF8 also have separate functions in macrophages.

### Regulation of the innate immune responses by IRF4

To compare the impact of IRF4 and IRF8 upon TLR signaling, Tot2-derived macrophages generated by the transduction of either IRF4 or IRF8 were stimulated with the TLR4 ligand lipopolysaccharide (LPS) or the TLR9 ligand CpG DNA. The induction of *Il12b* and *Il6* mRNAs was then measured. Consistent with the known role of IRF8 in *Il12b* transcription and TLR9 signaling, macrophages generated via IRF8 displayed much higher induction of *Il12b* upon both LPS and CpG treatment, and of *Il6* upon CpG treatment, compared with those generated by IRF4, although the latter cells showed low but measurable responses despite the lack of IRF8 expression (Figure 4B). Surprisingly, the induction of *Il6* upon LPS treatment was higher in IRF4-transduced macrophages than in IRF8-transduced macrophages. The empty virus-transduced Tot2 cells did not show any induction of either gene. These results suggest that IRF4 may have a positive role, in addition to its previously reported negative role [18], [19], in TLR signaling and/or the transcriptional regulation of cytokine genes.

As a possible mechanism, we found that both IRF4 and IRF8 strongly induce *Irf5* transcripts in Tot2 cells (Figure 2A). It has been shown that IRF5 binds to MyD88 upon recognition of various TLR stimuli, and then activated IRF5 functions as a direct transcriptional activator of multiple cytokine genes including *Il6* and *Il12b* [20]. Thus, IRF5 may, in part, mediate the positive effects of IRF4 and IRF8 on the innate immune responses in macrophages.

### IRF4 inhibits neutrophil differentiation

**Figure 5:**
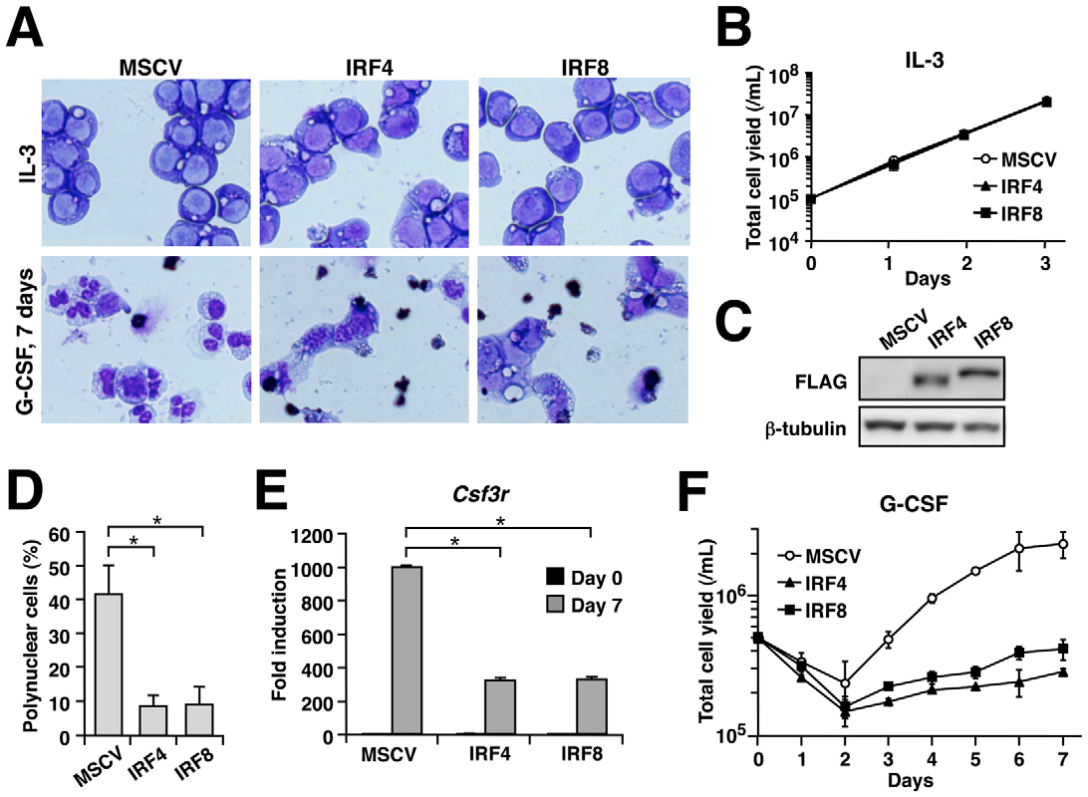
Inhibition of neutrophil differentiation by IRF4. (A) Wright-Giemsa staining of 32Dcl.3 cells transduced with empty MSCV-puro, MSCV-IRF4FLAG-puro or MSCV-IRF8FLAG-puro and cultured in the presence of IL-3 (upper panels) or G-CSF (for 7 days, lower panels). (B) Immunoblotting analysis of FLAG-tagged IRFs. β-tubulin expression is shown as a loading control. (C) Cell growth curves in the presence of IL-3. Data are expressed as mean ± standard deviation of triplicate determinations. (D) Proportions of cells showing the morphologic characteristics of mature granulocytes. *P<0.01 (Student's *t*-test). (E) *Csf3r* mRNA expression levels after 7 days of treatment of G-CSF. The expression levels were determined by qRT-PCR using the ΔΔCT method (mean ± standard deviation). Data are representative of two independent experiments with similar results. *P<0.01 (Student's *t*-test). (F) Viable cell yields during treatment with G-CSF. Data are expressed as mean ± standard deviation of three independent experiments.

We next sought to determine whether IRF4 has an ability to regulate neutrophil differentiation in a similar manner to IRF8. To this end, we employed 32Dcl.3 myeloid progenitor cells, which differentiate towards neutrophils when the supplemented cytokine is switched from IL-3 to G-CSF. In the presence of IL-3, the transduction of IRF4 or IRF8 did not affect cell viability or the proliferation of 32Dcl.3 cells (Figure 5A and C). In empty vector-transduced control cells, a 7-day culture with G-CSF showed morphological changes typical of neutrophils such as band/segmented nuclei in approximately 40% of the cells, whereas in cells transduced with IRF4 or IRF8, the same G-CSF treatment resulted in the appearance of mostly immature intermediate cells (Figure 5A and D). The expression levels of the introduced IRF4 and IRF8 were comparable (Figure 5B) and qRT-PCR analysis of the IRF4 or IRF8-transduced cells revealed that the induction of the gene encoding G-CSF receptor (*Csf3r*) upon G-CSF treatment was three-fold lower than in the control cells (Figure 5E). Indeed, the growth response to G-CSF was approximately seven-fold less than that of the control cells (Figure 5F). These results indicate that both IRF4 and IRF8 have an ability to inhibit neutrophil growth and differentiation.

### A more severe CML-like disease in mice doubly deficient for *Irf4* and *Irf8*

**Figure 6:**
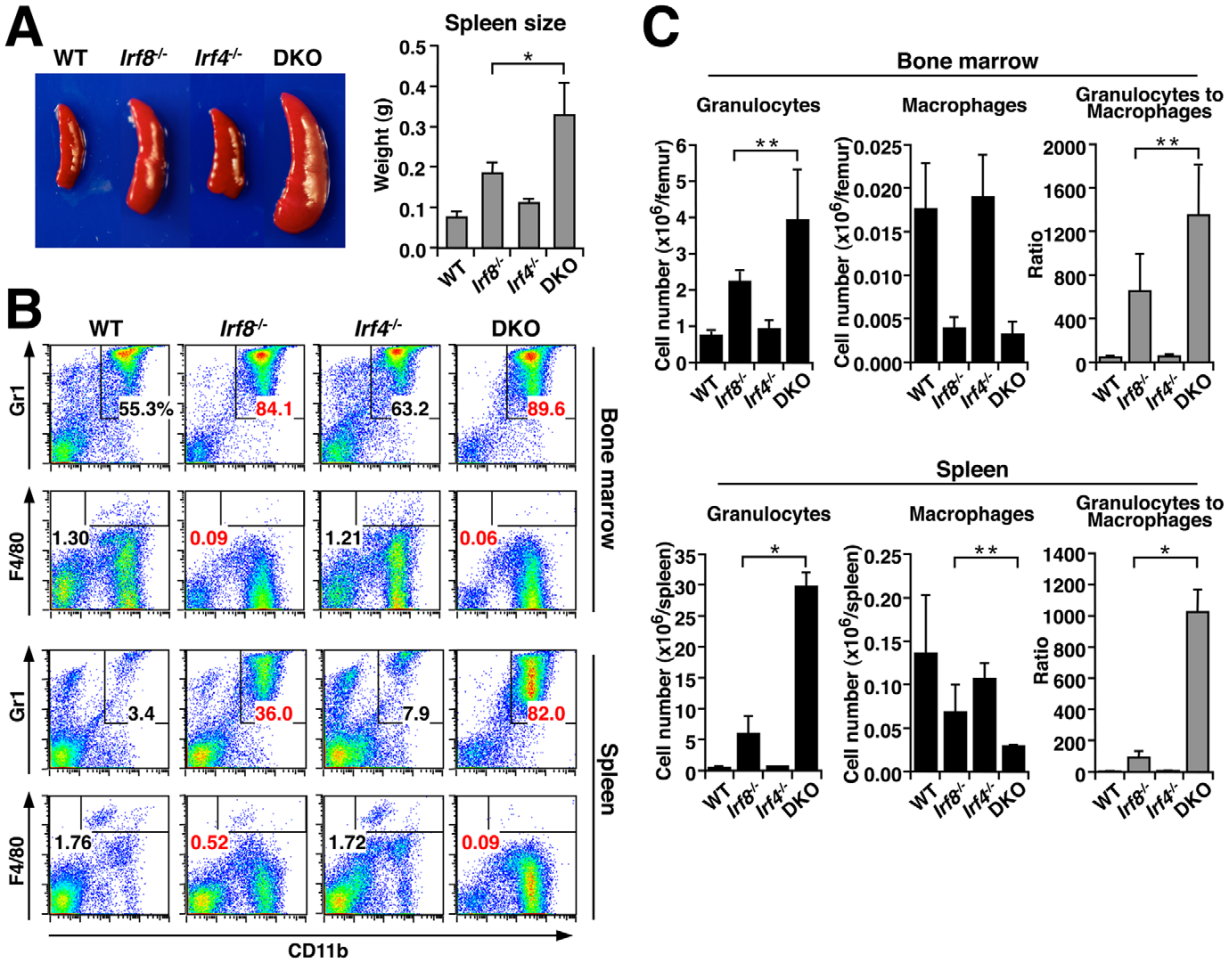
A CML-like disease in mice deficient for *Irf8* and *Irf4*. (A) Spleens (left panel) and spleen weight of WT, *Irf8* ^-/-^, *Irf4* ^-/-^, and DKO mice. Values are mean ± standard deviation from measurements of 5 to 6 spleens of each genotype. *P<0.01 (Student's *t*-test). (B) Flow cytometric analysis of granulocytes (CD11b^+^ Gr1^+^) and macrophages (F4/80^+^) in bone marrow cells (upper panels) and splenocytes (lower panels). Numbers indicate the percentages of granulocytes and macrophages. Data are representative of three independent experiments with similar results. (C) The absolute numbers of granulocytes and macrophages per femur (upper part) or spleen (lower part). The ratios of granulocytes to macrophages are shown in the right panels. Values are mean ± standard deviation from 3 to 5 mice of each genotype. *P<0.01 and **P<0.05 (Student's *t*-test).

To gain insight into the role of endogenous IRF4 in myeloid cell development, we analyzed mice that are deficient in *Irf8*, *Irf4* or both (*Irf8* ^-/-^, *Irf4* ^-/-^ or DKO, respectively). Splenomegaly, known to occur in *Irf8^-/-^* mice [8], was not observed in *Irf4* ^-/-^ mice at the age examined (7–9 weeks). However, DKO mice exhibited a more severe splenomegaly than *Irf8* ^-/-^ mice (Figure 6A), indicating a possible role of IRF4 that might be “hidden” if *Irf8* is intact. Flow cytometric analysis of splenocytes and bone marrow cells revealed that the increase in both the percentages and absolute numbers of CD11b^+^ Gr1^+^ granulocytes, known to occur in *Irf8* ^-/-^ mice, was markedly augmented in DKO mice, whilst *Irf4* ^-/-^ mice did not show an obvious increase (Figure 6B and C). On the other hand, the F4/80^+^ macrophage counts, known to decrease in *Irf8* ^-/-^ mice, were more severely diminished in DKO mice. Accordingly, the ratios of the granulocyte to macrophage numbers in the spleens of WT, *Irf8* ^-/-^, *Irf4* ^-/-^, and DKO mice were 3.3, 88.1, 6.0, and 1023.5, respectively (Figure 6C). Taken together, our data reveal that DKO mice exhibit a more severe myeloid development abnormality resembling CML compared with *Irf8* ^-/-^ mice, in which there is a disproportionate expansion of granulocytes at the expense of monocytes/macrophages. After we completed this work, Ren and colleagues also reported the expansion of granulocytes in *Irf4* ^-/-^ *Irf8* ^-/-^ mice [21]. In addition, the authors investigated the long-term fate of the mutant mice to show cooperative tumor suppressive functions for IRF4 and IRF8 in both myeloid and lymphoid cells.

### Expression of *Irf4* and *Irf8* mRNAs at various differentiation stages in hematopoietic cells

**Figure 7:**
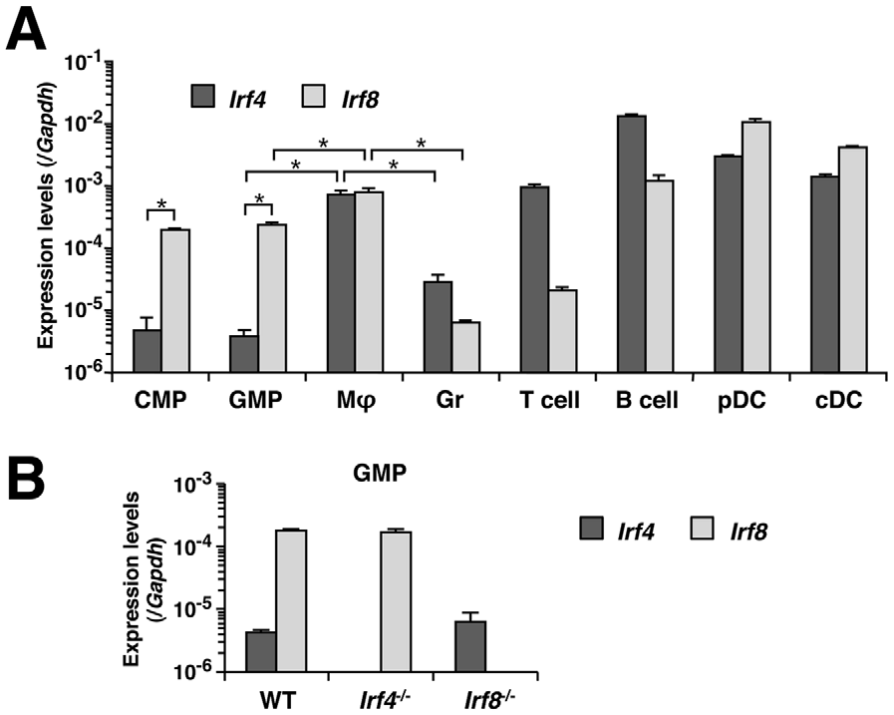
*Irf4* and *Irf8* transcript levels in myeloid and other hematopoietic cells. Expression levels of endogenous *Irf4* and *Irf8* mRNAs in common myeloid progenitors (CMPs), granulocyte/macrophage progenitors (GMPs), macrophages (MΦ), granulocytes (Gr), T cells, B cells, pDCs, and cDCs. Two to 10 mice were used to obtain RNA from each cell type. The pooled RNAs were analyzed in triplicate by qRT-PCR (mean ± standard deviation) using the standard curve method. Cells from WT mice (A) or mutant mice (B) were analyzed. *P<0.01 (Student's *t*-test).

To better understand why the loss of *Irf4* alone does not cause obvious abnormalities in myeloid cell development, whilst DKO mice show more severe defects than *Irf8* ^-/-^ mice, we examined the expression of endogenous *Irf4* and *Irf8* in CMPs, GMPs, macrophages and granulocytes, along with several other types of hematopoietic/immune cells. Whereas *Irf8* was clearly expressed in CMPs and GMPs, the expression of *Irf4* was very low in both of these progenitors (Figure 7A). This result provides a basis of the role of IRF8 being predominant and that of IRF4 becoming visible only when IRF8 is absent in myeloid progenitor cells. The *Irf4* transcript levels in *Irf8* ^-/-^ GMPs were found to be comparable with those in WT GMPs, indicating that it is unlikely that IRF8 functions as an IRF4 inducer (Figure 7B). Consistent with the common functions of these IRFs in stimulating and inhibiting the differentiation of macrophages and granulocytes, respectively, the expression of both *Irf4* and *Irf8* was found to be increased in macrophages but decreased to very low levels in granulocytes (Figure 7A).

## Discussion

We demonstrate in our current study that IRF4 has similar functions to IRF8 in the regulation of differentiation and growth in myeloid cells. Moreover, our present data suggest that each of these IRFs also has separate functions in macrophages.

*In vitro* differentiation experiments showed that IRF4, like IRF8, inhibits myeloid cell growth, and tunes the balance of lineage selection in myeloid progenitor cells by stimulating macrophage differentiation whilst inhibiting neutrophil differentiation. The results of reporter and ChIP assays revealed that IRF4 directly targets the IECS to activate the transcription of macrophage-related genes, clearly indicating that IRF4, as well as IRF8, functions intrinsically in myeloid cells. In support of these *in vitro* findings, *Irf8* ^-/-^ *Irf4* ^-/-^ mice display more severe CML-like symptoms than *Irf8* ^-/-^ mice. However, mice singly deficient in *Irf4* did not show any obvious abnormalities in their granulocyte and macrophage counts. One possible reason for this is the differential expression levels of IRF4 and IRF8 in myeloid progenitors (CMPs and GMPs). We observed by qRT-PCR that the expression level of *Irf8* in CMPs and GMPs is far higher than that of *Irf4*, whilst both IRF genes were clearly expressed in macrophages and at very low levels in granulocytes. We propose that during lineage selection in GMPs i.e. differentiation into macrophages or granulocytes, the role of IRF8 predominates but IRF4, expressed at a low level, can partially compensate for the absence of IRF8.

The activities of IRF4 and IRF8 in myeloid cells are not fully equivalent however. Whereas many macrophage-related genes such as *Ctsc*, *Cst3*, *Csf1r*, *Msr1*, *Prdm1*, and *Irf5* are induced both by IRF4 and IRF8, several genes were found to be induced by only one of the two IRFs. Specifically, IRF4 induces *Mmp12* and *Mrc1*, whilst only IRF8 induces *Itgae*. The specific induction of *Mmp12* and *Itgae* is reminiscent of a similar observation made previously in dendritic cells [16]. Mannose receptor is an M2 macrophage-related molecule [22] (see also below). Global gene expression profiling by microarray analysis supports the presence of both common and specific downstream genes for IRF4 and IRF8 (A.N., M.Y. and T.T., unpublished results). These results suggest that they have both common and specific activities, which are likely to confer basic features and functional diversity, respectively, upon macrophages.

It has been reported that IRF4 and IRF8 play distinct roles in innate immune responses. For example, IRF4 and IRF8 have been shown to affect the TLR-MyD88 pathway negatively and positively, respectively [18], [19], [23], [24]. Only IRF8 is induced by IFN-γ, the cytokine once referred to as the macrophage-activating factor, and participates in the transcriptional activation of IL-12p40 [25], [26]. Our present data indeed demonstrate that IRF8 is far more potent than IRF4 in supporting TLR9 signaling and inducing the *Il12b* gene. Whilst the accumulating evidence suggests that IRF8 is essential for M1 macrophage polarization via the induction of IL-12p40, Akira and colleagues have reported very recently that IRF4 is essential for the M2 polarization [27]. We also observed that IRF4 but not IRF8 induces *Mrc1*, that encodes an M2 macrophage-related molecule [22]. Yet, our current study indicates a more complex picture of the role played by IRF4; it stimulates LPS (but not CpG) induction of the gene encoding the M1 cytokine IL-6 more strongly than IRF8 [22]. The “positive” effects of IRF4 on the innate immune responses may be mediated, at least in part, by the induction of *Irf5* because IRF5 is essential for the signaling that occurs through various TLRs and is implicated in the transcriptional activation of multiple cytokine genes including *Il6* [20]. However, the induction of *Irf5* occurs also via IRF8, suggesting that this represents a previously unrecognized “common and positive” effect of IRF4 and IRF8 on the innate immune responses. The mechanism underlying the “specific and positive” potential of IRF4 requires further investigation. Because the heterogeneity of macrophages has been progressively uncovered, it will be important to further investigate the usage of IRF4 and IRF8 in different macrophage subsets.

It is noteworthy that IRF4 and IRF8 are essential for the development of all professional antigen presenting cells (APCs) including macrophages (this study), DCs and B cells [14], [16], [28]. In these APCs, IRF4 typically stimulates Th2 responses, whilst IRF8 induces Th1 responses [16], [27], [29], [30]. Furthermore, IRF4 and IRF8 also play a role in T cells where IRF4 is required for Th2, Th17 and Th9 responses, whereas IRF8 inhibits Th17 responses [31], [32], [33], [34]. Hence, the similar but distinct transcription factors IRF4 and IRF8, which are present only in vertebrates, make a critical contribution to diverse immune responses by acting in professional APCs as well as in T cells, both of which are the hallmark of the vertebrate immune system.

It has been shown previously that not only *IRF8* but also *IRF4* transcript levels are significantly diminished in human CML patients [11], [35], [36]. Moreover, *IRF8* and *IRF4* expression correlates with the cytogenetic response to IFN-α. Importantly, these observations are not secondary phenomena due to the expansion of neutrophils that do not express these IRFs, because the expression of *IRF8* and *IRF4* is also diminished in sorted B and T cells, respectively. The Bcr/Abl kinase inhibitor imatinib has replaced IFN-α as the first-line therapy for CML. However, this inhibitor cannot effectively eliminate leukemic stem cells [37], [38], and if the drug is discontinued, most patients eventually relapse. The next generation of therapies for CML is thus eagerly awaited. Notably, it is well established that CML cells are highly sensitive to T cell-mediated immunity [39]. Naïve T cells are activated by professional APCs, particularly DCs and macrophages, whose differentiation and function are cooperatively regulated by IRF8 and IRF4, as revealed by the current and previous studies. In this regard, it will be interesting to more closely examine whether CML patients have any defects in the development and function of their professional APCs. Thus, the lack of IRF8 and IRF4 expression is likely to be critically involved in the pathogenesis of human CML, and seeking a way to restore the expression and function of these IRFs could be a powerful new approach to the improvement of CML therapy.
